# From Wolf-Hirschhorn syndrome to NSD2 haploinsufficiency: a shifting paradigm through the description of a new case and a review of the literature

**DOI:** 10.1186/s13052-022-01267-w

**Published:** 2022-05-12

**Authors:** Luisa Cortellazzo Wiel, Irene Bruno, Egidio Barbi, Fabio Sirchia

**Affiliations:** 1 University of Trieste, Piazzale Europa, 1, 34127 Trieste, Italy; 2 Institute for Maternal and Child Health – IRCCS Burlo Garofolo, Trieste, Italy; 3 Department of Molecular Medicine, University of Pavia, Pavia, Italy; 4 IRCCS Mondino Foundation, Pavia, Italy

**Keywords:** Wolf-Hirschhorn syndrome, NSD2, growth restriction, intellectual disability, facial gestalt, case report

## Abstract

**Background:**

Wolf-Hirschhorn syndrome (WHS) is a well-defined disorder, whose core phenotype encompasses growth restriction, facial gestalt, intellectual disability and seizures. Nevertheless, great phenotypic variability exists due to the variable extent of the responsible 4p deletion. In addition, exome sequencing analyses, recently identified two genes, namely *NSD2* and *NELFA*, whose loss-of-function variants contribute to a clinical spectrum consistent with atypical or partial WHS.

The observation of patients exhibiting clinical features resembling WHS, with only mild developmental delay and without the typical dysmorphic features, carrying microdeletions sparing *NSD2*, has lead to the hypothesis that *NSD2* is responsible for the intellectual disability and the facial gestalt of WHS. While presenting some of the typical findings of WHS (intellectual disability, facial gestalt, microcephaly, growth restriction and congenital heart defects), *NSD2*-deleted children tend to display a milder spectrum of skeletal abnormalities, usually consisting of clinodactyly, and do not exhibit seizures.

We describe the clinical picture of a child with WHS due to a de novo mutation of NSD2 and discuss the clinical and diagnostic implications.

**Case presentation:**

A 6-year-old boy was evaluated for a history of intrauterine growth restriction, low birth weight, neonatal hypotonia, and psychomotor delay. No episodes of seizure were reported. At physical examination, he displayed marphanoid habitus, muscle hypotrophy and facial dysmorphisms consisting in high frontal hairline, upslanting palpebral fissures and full lips with bifid ugula. Cryptorchidism, shawl scrotum, mild clinodactyly of the right little finger and bilateral syndactyly of the II and III toes with sandal gap were also noted. The radiographic essay demonstrated delayed bone age and echocardiography showed mild mitral prolapse. Whole genome sequencing analysis revealed a heterozygous de novo variant of *NSD2* (c.2523delG).

**Conclusions:**

Full WHS phenotype likely arises from the cumulative effect of the combined haploinsufficiency of several causative genes mapping within the 4p16.3 region, as a contiguous genes syndrome, with slightly different phenotypes depending on the specific genes involved in the deletion.

When evaluating children with pictures resembling WHS, in absence of seizures, clinicians should consider this differential diagnosis.

## Background

Wolf-Hirschhorn syndrome (WHS) is a well-defined disorder due to variable size-deletions of the chromosomal region 4p16.3, characterized by a clinical picture encompassing growth restriction, developmental delay, microcephaly, congenital hypotonia and major malformations, including midline, heart, renal and skeletal defects, along with the typical facial gestalt, consisting of the so called “Greek warrior helmet” appearance (high forehead, continuing to a wide nasal bridge, with short philtrum, high arched eyebrows, hypertelorism, and micrognathia). Seizures occur in nearly all affected patients within the age of 3 years and complicate the management, acting as a significant prognostic factor for the final degree of intellectual disability.

In front of the great phenotypic variability of WHS, depending mostly on the extent of the 4p deletion, the core WHS phenotype is conventionally defined by the association of intellectual disability, growth delay, facial gestalt and seizures [1]. Thus, two minimal critical regions responsible for WHS (WHSCR) have been identified, corresponding to the smallest region, whose haploinsufficiency determines the core phenotype [2–4].

More recently, exome sequencing analyses identified two genes within the WHSCR, whose loss-of-function variants contribute to a clinical spectrum consistent with atypical or partial WHS: WHS candidate gene 1 (*WHSC1*), also known as Nuclear receptor-binding Set Domain-protein 2 (*NSD2*), contained only partly within the WHSCR [5], and WHS candidate gene 2 (*WHSC2*), also known as Negative Elongation Factor Complex Member A (*NELFA*), entirely contained within the WHSCR [6].

We describe a patient with a de novo variant of *NSD2* and discuss the clinical implications.

## Case presentation

The proband was a 6-year-old boy, born at 34 weeks of gestation by cesarean section from healthy, non-consanguineous parents. Gestation was complicated by intrauterine growth restriction (IUGR) and the baby displayed low birth weight. The neonatal period was characterized by hypotonia, followed by psychomotor delay. No episodes of seizure were reported.

**Fig. 1:**
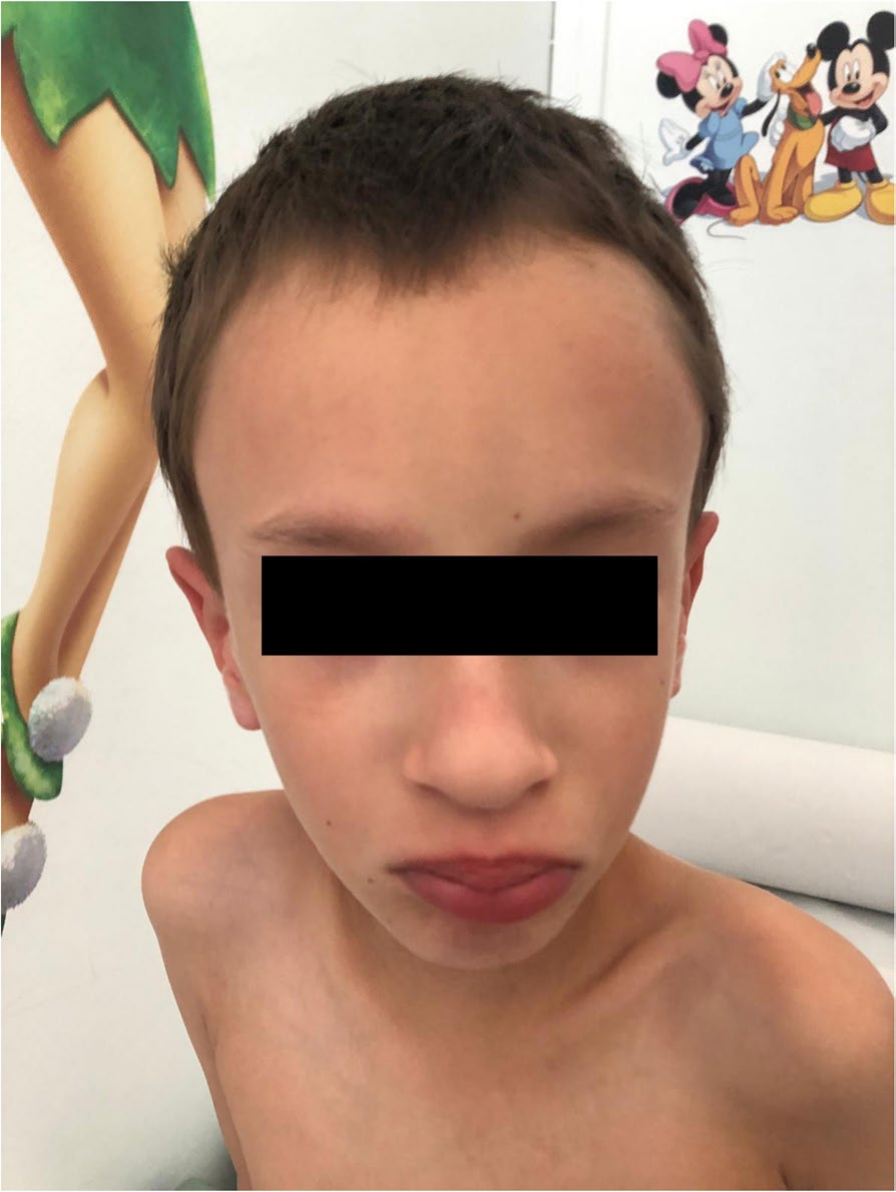
Patient’s facies, characterized by high frontal hairline, upslanting palpebral fissures and full lips

At physical examination, he displayed marphanoid habitus, muscle hypotrophy and facial dysmorphisms consisting in high frontal hairline, upslanting palpebral fissures and full lips with bifid ugula (Fig. 1). Cryptorchidism, shawl scrotum, mild clinodactyly of the right little finger, bilateral syndactyly of the II and III toes with sandal gap and a small café-au-lait spot on dorsum were also noted. The radiographic essay demonstrated delayed bone age and echocardiography showed mild mitral prolapse.

Both single nucleotide polymorphism-arrays and next-generation intellectual disability gene panel proved negative. Whole genome sequencing analysis revealed a heterozygous de novo variant of *NSD2* (c.2523delG).

## Discussion and conclusions

*NSD2* acts as a histone methyltransferase, responsible for the methylation of HEK36, thus explaining the occurrence of developmental delay in carriers of *NSD2* variants, in light of the crucial role of histones modification in brain development. Of note, the description of two patients with intact *NSD2*, exhibiting clinical features resembling WHS but only mild developmental delay [7], has lead to the assumption that the haploinsufficiency of *NSD2* is responsible for the developmental delay, typically observed in WHS patients; this hypothesis has been further supported by the documentation of a higher degree of developmental delay in patients with disrupted *NSD2*, compared with those with the intact gene [8, 9]. Autism spectrum disorder has been reported in eight *NSD2*-haploinsufficient children [10, 11]. Moreover, deletions of *NSD2* are considered responsible for the facial gestalt of WHS, in light of the observation of non-specific findings consistent with WHS (growth and developmental delay) but without the typical dysmorphic features, in several patients with microdeletions sparing *NSD2* [12, 13].

Hence, the clinical spectrum of *NSD2* deletion encompasses: prenatal and postnatal growth restriction [14], microcephaly, developmental delay [15], congenital heart defects and several phenotypic traits, including hypertelorism, upward-slanting palpebral fissures, prominent nasal bridge, abnormal teething and micrognathia. Cleft palate has been described in fourteen patients [16–21]. Compared to WHS patients, *NSD2*-deleted children tend to display a milder spectrum of skeletal abnormalities, usually consisting of clinodactyly [22]. Table 1 summarizes the previously reported cases of NSD2 haploinsufficiency. Remarkably, seizures are not usually part of the clinical spectrum of *NSD2* variants.

**Table 1:** Summary of previous published cases of NSD2 haploinsufficiency

	Zollino et al.,	Rauch et al.,	Zollino et al.,	Van Buggenhout et al.,	Rodrìguez et al.,	Maas et al.,	Izumi et al.,	Okamoto et al.,	Andersen et al.,
	Am J Med Genet 2000	Am J Med Genet 2001	Am J Hum Genet 2003	J Med Genet 2004	Am J Med Genet 2005	J Med Genet 2008	Am J Med Genet 2010	Am J Med Genet 2013	Eur J Med Gen 2014
				5 patients					3 patients
Age at last observation (years)		5	1	5.6 to 13.3	4		2.8	2	2 to 11
Genetic finding	12 patients with > 5 Mb deletion and 3 patient s with < 5 Mb deletion	191.5 kb deletion	1.9 Mb deletion	> 2 Mb deletion	1.9 Mb deletion	8 patients with > 5 Mb deletion and 13 patients with ≤5 Mb deletion	1.3 Mb deletion	109 kb deletion	60 to 377 kb deletion
IUGR	13/15	–	+	3/5	+	18/21	+	+	2/3
SGA	13/15	–	–	3/5	+	15/20	+	+	2/3
Postnatal growth retardation	15/15	+	+	5/5	+	16/21	+	+	1/3
Microcephaly	14/15	–	–	2/5	–	19/20	–	+	0/3
Craniofacial	15/15	+	+	5/5	+	20/21	+	+	3/3
High/broad forehead	N/A	+	+	1/5	–	17/18	–	+	3/3
Frontal bossing	N/A	–	+	0/5	+	0/21	–		N/A
Bitemporal narrowing	N/A	+	–	0/5	–	0/21	–	–	2/3
Prominent glabella	N/A	+	+	0/5	–	14/19	–	+	1/3
High arched eyebrows	N/A	+	–	0/5	–	0/21	–	+	1/3
Hypertelorism	N/A	+	+	0/5	–	16/19	–	+	3/3
Epicanthus	N/A	+	–	0/5	+	7/17	–	+	2/3
Prominent eyes	N/A	+	+	1/5	–	0/21	–	+	2/3
Downslanting palpebral fissures	N/A	–	–	1/5	–	0/21	+	+	0/3
Abnormal ears	N/A	+	+	1/5	+	17/20	–	+	2/3
Broad nasal bridge	N/A	+	–	4/5	+	13/19	+	+	3/3
Short philtrum	N/A	+	–	1/5	+	16/19	+	+	2/3
Downturned corners of the mouth	N/A	+	+	0/5	–	14/19	–	+	0/3
Micrognathia	N/A	+	–	0/5	–	15/20	+	+	3/3
Cleft lip/cleft palate	6/15	–	–	1/5	–	3/19	–	–	0/3
Hypotonia	15/15	–	+	0/5	+	13/19	+	–	2/3
Feeding difficulties	N/A	+	–	1/5	–	N/A	–	+	3/3
Seizures	14/15	–	+	2/5	+	15/21	+	–	0/3
Developmental delay	15/15	+	–	5/5	+	21/21	+	+	3/3
Abnormal behaviour	N/A	ADHD	–	N/A	N/A	N/A	N/A	ADHD, aggressiveness	ADHD 1/3
CNS structural abnormalities	N/A	N/A	–	Sacral dimple 4/4; delayed myelinisation 1/1	Sacral dimple	Sacral dimple 15/17	N/A	–	Distal ventral chordae 1/1
Hearing loss	N/A	+	–		N/A	N/A	N/A	N/A	
Ophtalmological abnormalities	Iris coloboma 1/15	–	N/A	N/A	N/A	N/A	N/A	N/A	0/1
Cardiac features	9/15	–	–	N/A	–	ASD 2/19; AVSD 1/19; VSD 1/11; pulmonary stenosis 1/11	N/A	–	Left aortic arch, retroesophageal subclavian artery 1/3
Urinary tract malformations	Renal hypoplasia 3/15; hydronephrosis 3/15; renal fusion 1/15; hypospadias 5/15	–	–	Hypospadias 1/1; inguinal hernia 1/1	Bilateral pyelectasia	Left kidney duplication 1/18; VUR 8/18; hypospadias 8/18; right cryptorchidism 1/18; clitoridomegaly 1/18; ventrally spaced anus 1/8	N/A	–	VUR 1/3; left pyelectasia, cryptorchidism 1/3
Congenital diaphragmatic hernia	0/15	–	–	1/5	–	0/21	–	–	0/3
Skeletal abnormalities	N/A	Clinodactyly of V fingers, cutaneous syndactyly of I and III toes	–	Hyperkyphosis 2/5; small hands and feet 1/1	–	Scoliosis/hyperkyphosis 7/19; club feet 3/17	Small nails	–	0/3

	Yang et al.,	Lozier et al.,	Callaway et al.,	Derar et al.,	Bernardini et al.,	Boczek et al.,	Barrie et al.,	Jiang et al.,	Hu et al.,	Zanoni et al.,
	Chinese Medical Journal 2016	Hum Genet 2018	J Pediatr Genet 2018	Genet in Med 2018	Am J Med Genet 2018	Am J Med Genet 2018	Cold Spring Harb Mol Case Stud 2019	BMC Med Genet 2019	BMC Med Genom 2020	Genet in Med 2021
				2 patients	3 patients		3 patients			
Age at last observation (years)		1.3	Stillborn	2.8 to 5	3 to 7.2	3	2.2 to 5	12	10.5	
Genetic finding	10 patients with > 2 Mb deletion	Pathogenic SNV	19 Mb deletion	Pathogenic SNV	68 to 166 kb deletion	Pathogenic SNV	Pathogenic SNV	Pathogenic SNV	Pathogenic SNV	18 patients with pathogenic SNV
IUGR	5/10	–	+	2/2	2/3	+	3/3	+	+	7/17
SGA	5/10	–	N/A	N/A	3/3	+	2/2	+	+	1/18
Postnatal growth retardation	10/10	+	N/A	2/2	3/3	+	2/3	+	+	16/17
Microcephaly	0/10	+	N/A	2/2	0/3	+	2/3	–	+	1/18
Craniofacial	10/10	+	N/A	2/2	3/3	+	3/3	+	+	17/17
High/broad forehead	10/10	+	N/A	0/2	3/3	–	1/3	–	–	8/17
Frontal bossing	0/10	–	N/A	0/2	N/A	–	0/3	–	–	2/17
Bitemporal narrowing	0/10	–	N/A	0/2	2/3	–	0/3	–	–	0/18
Prominent glabella	10/10	–	N/A	0/2	0/3	–	0/3	+	–	0/18
High arched eyebrows	10/10	–	N/A	1/2	3/3	+	0/3	+	+	2/17
Hypertelorism	10/10	+	N/A	1/2	2/3	+	0/3	+	+	6/17
Epicanthus	0/10	+	N/A	0/2	3/3	–	0/3	+	+	2/17
Prominent eyes	9/10	–	N/A	0/2	3/3	–	0/3	–	–	0/18
Downslanting palpebral fissures	0/10	–	N/A	0/2	0/3	–	0/3	–	–	0/18
Abnormal ears	2/10	+	N/A	2/2	2/3	+	1/3	–	+	8/17
Broad nasal bridge	10/10	+	N/A	2/2	2/3	+	1/3	+	–	4/17
Short philtrum	9/10	–	N/A	2/2	2/3	–	0/3	+	–	2/17
Downturned corners of the mouth	5/10	–	N/A	2/2	2/3	–	0/3	+	–	0/18
Micrognathia	4/10	–	N/A	2/2	1/3	–	0/3	+	+	3/17
Cleft lip/cleft palate	2/0	–	+	0/2	1/3	–	0/3	–	–	0/18
Hypotonia	9/10	+	N/A	2/2	2/3	+	3/3	+	–	12/17
Feeding difficulties	3/10	–	N/A	2/2	1/3	–	0/3	+	–	9/18
Seizures	8/10	–	N/A	0/2	0/3	–	0/3	–	–	2/17
Developmental delay	10/10	+	N/A	2/2	3/3	+	3/3	+	+	13/17
Abnormal behaviour	N/A	N/A	N/A	0/2	ADHD 1/3	Anxiety, hyperactivity, aggressiveness	Autism 1/1	N/A	N/A	Autism 7/17; ADHD 6/17; Aggressiveness 2/17; Anxiety 2/17
CNS structural abnormalities	N/A	N/A	N/A	0/2	Subependymal and intarathalamic hyperechogenic spots 1/3	Sacral dimple	Isolated 4 mm subependymal gray matter heterotopia 1/2	–	N/A	T2 hyperintensity and volume loss of the periatrial white matter 1/10; 8 mm pineal cyst, non-specific mild diffuse T2/FLAIR signal hyperintensity in the bilateral parietal and occipital white matter, small thoracic spinal cord syrinx 1/10; few small areas of gliosis in the frontal and periatrial area 1/10; thin corpus callosum, white matter lesions 1/10
Hearing loss	N/A	N/A	N/A			N/A		N/A	N/A	0/16
Ophtalmological abnormalities	N/A	N/A	N/A	0/2	Myopia 1/1	N/A	Myopia 1/1	N/A	N/A	Refraction defects 4/18; strabismus 2/18; bilateral keratoconus, retinitis pigmentosa, optic atrophy, corneal transplant 1/18
Cardiac features	ASD 3/8; pulmonary stenosis 2/8	N/A	N/A	0/2	Partial AVCD 1/2; mitral valve prolapse 1/2	N/A	N/A	–	–	Mild pulmonary artery stenosis 1/18; small PFO 1/18; interrupted aortic arch, VSD 1/18
Urinary tract malformations	1/10	Rotation of right kidney	N/A	0/2	0/3	N/A	Bilateral renal hypoplasia, CKD 1/2	–	N/A	Left renal agenesis 1/18; left hydronephrosis and hypospadias 1/18; bilateral cryptorchidism 1/18; congenital bilateral inguinal hernia 1/18
Congenital diaphragmatic hernia	0/10	–	+	0/2	0/3	–	0/3	–	–	0/18
Skeletal abnormalities	1/10	Clinodactyly of V fingers	N/A	0/2	Mild scoliosis, bilateral pes cavus, syndaktyly II-III toes 1/3	–	0/3	Clinodactyly of V fingers	–	8/18 (pectus excavatum, scapulae alatae, mild scoliosis, prominent knees, flat feet, broad forefeet, bilateral clinodactyly of the V toe 1/18; 11 ossified ribs and 6 non rib-bearing lumbar vertebrae 1/18; severe arthrosis of wrist and knee 1/18; clinodactyly of V fingers 3/18; bilateral cutaneous incomplete syndactyly of II and III toes 2/18; pes planus 2/18)

*Abbreviations*: *SNV* single nucleotide variant, *IUGR* intrauterine growth restriction, *SGA* small for gestational age, *CNS* central nervous system, *N/A* not assessed, *ADHD* attention deficit and hyperactivity disorder, *ASD* atrial septum defect, *AVSD* atrioventricular septum defect, *VSD* ventricular septum defect, *AVCD* atrioventricular canal defect, *PFO* patent foramen ovale, *VUR* vescicoureteral reflux, *CKD* chronic kidney disease

*LETM1* (Leucine zipper/EF-hand containing transmembrane), involved in calcium signaling and mapping within the WHSCR, had been previously identified as responsible for seizures. However, this assumption has recently been questioned by the observation of the occurrence of seizures in children carrying terminal 4p deletions sparing *LETM1*, and of the lack of seizure in individuals with interstitial deletions including *LETM1*, but preserving a relatively large terminal 4p segment [23]: these observations suggest that the haploinsufficiency of *LETM1* alone may not be sufficient in causing seizures, which would rather result from the effect of additional candidate genes [24].

Remarkably, the recurrence risk of *NSD2* variants is 50% and must be taken into account when counseling families of affected individuals.

In conclusion, full WHS phenotype probably arises from the cumulative effect of the combined haploinsufficiency of several causative genes mapping into the 4p16.3 region, as a contiguous genes syndrome, with slightly different phenotypes depending on the specific genes involved in the deletion [25].

*NSD2* haploinsufficiency is responsible of a distinctive entity, with clinical findings falling to some extent within the WHS phenotype, but not sufficient to allow a conclusive diagnosis of WHS.

When evaluating children with pictures resembling WHS, clinicians should bear this condition in mind as a possible differential diagnosis.

## Data Availability

Data sharing is not applicable to this article as no datasets were generated or analyzed during the current study.

## Abbreviations

WHS: Wolf-Hirschhorn syndrome
*WHSC1*: Wolf-Hirschhorn syndrome candidate gene 1
*NSD2*: Nuclear receptor-binding Set Domain-protein 2
*WHSC2*: Wolf-Hirschhorn syndrome candidate gene 2
WHSCR: Wolf-Hirschhorn syndrome Critical Region
*NELFA*: Negative Elongation Factor Complex Member A
*LETM1*: Leucine zipper/EF-hand containing transmembrane
